# The Effect of Neonicotinoids Exposure on *Oreochromis niloticus* Histopathological Alterations and Genotoxicity

**DOI:** 10.1007/s00128-022-03611-6

**Published:** 2022-09-18

**Authors:** Islam M. El-Garawani, Elsayed A. Khallaf, Alaa A. Alne-na-ei, Rehab G. Elgendy, Hassan M. Sobhy, Adel Khairallah, Heba M. R. Hathout, Farag Malhat, Amany E. Nofal

**Affiliations:** 1 Zoology Department, Faculty of Science, Menoufia University, Shebin El-Kom, 32511 Menoufia Egypt; 2 Department of Natural Resources, Faculty of African Postgraduate Studies, Cairo University, Giza, 12613 Egypt; 3 Department of Pesticide Residues and Environmental Pollution, Central Agricultural Pesticide Laboratory, Agriculture Research Center, Dokki, Giza, 12618 Egypt

**Keywords:** Neonicotinoids, Nile tilapia, Acetamiprid, Imidacloprid, Micronucleus, Toxicity

## Abstract

This study aimed to examine the side effects of selected neonicotinoids (Acetamiprid, Aceta, and Imidacloprid, Imid) on *Oreochromis niloticus* juveniles. The acute toxicity, Probit method, revealed an LC_50_ of 195.81 and 150.76 ppm for Aceta/96 h and Imid/72 h respectively. The fish were divided into three groups that were exposed, for 21 days (n = 5/replicate), to 1/10 of the LC_50_ of either neonicotinoids, however, the third was an unexposed control group. Results of erythrocytic micronucleus (MN), and nuclear abnormalities (NA) showed that Aceta and Imid exposure caused a significant (*p* < 0.05) increase in MN by ~ 2.2 and ~ 10 folds, respectively relative to control. NAs occurred at the order of kidney-shaped > budding > binucleated in Aceta, however, budding > binucleated > kidney-shaped was noticed in the Imid group. Histopathological changes in gills, liver, and muscles were observed significantly in both exposed groups with more severity in the Imid group. Collectively, Aceta and Imid have potential genotoxicity and histopathological alterations in *O. niloticus*.

Neonicotinoids are highly water-soluble organic insecticides commercialized in over 120 countries (Jeschke et al. 2011). In comparison to nicotine, neonicotinoids are nicotinic receptor stimulants that specifically interact with the insect's nicotinic acetylcholine receptor (nAChR) (Simon-Delso et al. 2015). They are categorized as nAChR competitive modulators by the Insecticide Resistance Action Committee (IRAC) (Malhotra et al. 2021). Because neonicotinoids are rapidly carried into surface water by leaching, percolation and runoff from agricultural regions, they pose a significant danger to environmental water quality, threatening non-target species and aquatic organisms (Solomon et al. 2013; Yi et al. 2019; Marins et al. 2021). Belonging to the chloronicotinyl neonicotinoids, imidacloprid and acetamiprid are two widely used insecticides. They have a broad insecticidal spectrum, excellent systemic and translaminar properties, and highly active residuals (Horowitz et al. 1998). Firstly, acetamiprid (Aceta) is extensively used in agriculture world-wide, mainly, to control lepidopteran and hemipteran pests in a variety of crops (Guedegba et al. 2019; Cossi et al. 2020). The second is imidacloprid pesticide (Imid) which is the most effective, widely and currently the best-selling neonicotinoid insecticide on rice paddies and other crops to control plant and leaf-hoppers, aphids, thrips, and insects (Demirci and Güngördü 2020; Naiel et al. 2020; El-Garawani et al. 2021a, b). Long-term exposure to water contaminants, even at low concentrations, can cause morphological, histological, and biochemical alterations in fish tissues, lowering the quality and marketability of the fish (Haredi et al. 2020). Tilapia is a Cichlidae genus that is widely distributed throughout Africa, South America, and the Middle and Far East (Kadry et al. 2015). In Egypt, Nile tilapia (*Oreochromis niloticus*) is an important commercial fish accounting for a large percentage of the overall catch each year along the Nile (Kadry et al. 2015). It is a popular aquaculture fish that can withstand a broad range of environmental conditions, including salinity and pollution, and has a low sensitivity to diseases (Tayel et al. 2008). In aquatic ecosystems with abundant accumulation of pesticides and related chemicals, fish organs such as liver, gills and muscles are usually the targets of pesticide accumulation leading to the highest injury (Stoskope 1993; Visoottiviseth et al. 1999; Haredi et al. 2020) due to their importance in the quality of fish farming and human consumption. Numerous studies revealed a variability of alterations affecting fish liver consequential to the pesticides' exposure (Visoottiviseth et al. 1999). Histopathological changes in *Oreochromis niloticus* induced by pesticides were monitored (Kan et al. 2012; Dim et al. 2015; Ansoar-Rodríguez et al. 2016). Ghaffar et al. (2020) reported that gill sections from fish treated with neonicotinoid pesticides exhibited secondary lamellae atrophy, lamellar epithelial pillar cell pyknosis, of primary and secondary lamellae congestion and degeneration in exposed *Labeo rohita* fish. Time-dependently, acetamiprid affected the histopathological structure of *C. mrigala* gills and liver (Ghayyur et al. 2021). However, gill and liver tissues were histopathologically affected by imidacloprid exposure (Ansoar-Rodríguez et al. 2016; Günal et al. 2020), in gonads (Lee 2003) of *O. niloticus*, and in the kidneys of *O. mossambicus* and *L. rohita* (Patel et al. 2016). In fish erythrocytes, the micronucleus (MN) test has been employed as an indicator of environmental mutagenesis (Al-Sabti and Metcalfe 1995; Sayed et al. 2016). Micronucleus (MN) and erythrocytic nuclear abnormalities (NA) assessment provides data on environmental quality, species health, genotoxicity, and possible risk (Al-Sabti and Metcalfe 1995; Mekkawy et al. 2011; Sayed et al. 2016). The elevated nuclear abnormalities were reported in *O. niloticus* erythrocytes exposed to deltamethrin insecticide (Kan et al. 2012), different concentrations of imidacloprid Ansoar-Rodríguez et al. (2015) and silver barb fish exposed to profenofos (Khan et al. 2018). The aim of this study was to comparatively evaluate the sub-lethal hazards of selected neonicotinoids (acetamiprid and imidacloprid) on exposed *Oreochromis niloticus* juveniles. The extent of toxicity was assessed in liver, gill and muscle tissues as histopathological changes in addition to erythrocytic micronuclei and nuclear abnormalities.

## Materials and Methods

The Fish Hatchery Station of Kafr El-Sheikh Governorate, Egypt, provided juveniles of *O. niloticus* (12.4 ± 3.4 g, 9.9 ± 1.7 cm). Aquaria with dechlorinated tap water (50 L) were used to acclimate the fish for 14 days. The constant aeration was performed using electric air pumps. Stable conditions of water were maintained at 20.66 ± 0.825°C, 6.88 ± 0.45 pH, 823 ± 40.1 µS/cm conductivity, 0.045 ± 0.025 mg/L of ammonia concentration. Fish were fed on a commercial diet (25% protein, Tag-elmlook Company, Kafr-El-Sheikh). Feeding was postponed by about 24 h prior to the start of the experiment.

Acetamiprid, (CAS:20180430005, Telfast 20 SP, 20%, Shandong United Pesticide Industry Co., Ltd., Shandong, China).

Imidacloprid, [1-(6-chloropyridin-3-ylmethyl)-*N*-nitroimidazolidin-2- /ylideneamine], was provided by PHARMA CURE CO. (CLAS 35% SC, CAS. 1811, Wady Al-Natron, Egypt) as a commercial form.

Determination of half lethal concentration (LC_50_) toxicity tests was carried out on fish (*n* = 10/group) exposed to six series of acetamiprid and imidacloprid concentrations (0, 50, 100, 150, 200, 250 and 300 ppm) using the Probit procedure (Finney 1952). The cumulative mortality was obtained at 24, 48, 72 and 96 h and represented by Probit regression for both insecticides. The 100% lethal concentration was recorded between 250 and 300 ppm for Aceta-96 h and Imid-72 h. Thus, the end point intervals for LC_50_ were selected as 96 h for Aceta and 72 h for Imid.

To assess the toxic effects of Aceta and Imid, three groups were created using five fish each. In dechlorinated tap water-contained glass aquaria, fish at a stocking density of 1.24 g/L were maintained. The first group served as a control and the second was exposed to Aceta 19.5 ppm (1/10 of LC_50_) and the third group was subjected to Imid at 15.0 ppm (1/10 of LC_50_). The experiment was prolonged for 21 days according to Al-Anazi et al. (2015). In order to maintain the nominal concentration of both insecticides throughout the experiment, water was renewed (100%) daily to avoid Imid and Aceta degradation based on the results of HPLC analysis. A quantitative analysis of Aceta and Imid was conducted using an Agilent 1260 series HPLC–DAD. HPLC separation was performed on a Zorbax XDB C18 (4.6 mm × 250 mm × 5 μm) column. Acetonitrile and water (70:30, v/v, isocratic conditions) were used as the mobile phase for elution of Aceta, while methanol and water (60:40, v/v, isocratic conditions) were used for elution of Imid. A flow rate of 0.8 mL/min was used. The column thermostat temperature was controlled at 35°C during analysis, and the detection was done at a wavelength of 246 and 260 nm for Aceta and Imid, respectively. The injection volume was 20 μL. Using samples spiked with the tested insecticides, the sensitivity and recovery were determined. The mean recovery in water samples for the tested insecticides ranged from 90% to 96%, with a relative standard deviation (RSD) of 5%–12.5%. Recovery rates and their relative standard deviation were acceptable. The limit of detection (LOD) for both insecticides (Aceta and Imid) was 0.1 ng/L. The reference standards of both compounds were run prior to analysis to check the performance of the column, peak height, resolution, and limits of detection. For each set of samples to be analysed, a solvent blank, a calibration standard of each tested insecticide and a procedural blank were run in sequence to check for contamination, peak identification and quantification. The reference standards of acetamiprid and imidacloprid were purchased from Dr. Ehrenstorfer (Augsburg, Germany) with purities greater than 99%. The water measurements of temperature, pH, conductivity and levels of oxygen and ammonia were parallel to those of acclimatization conditions. Experiments were done in triplicates.

The collection of blood samples was done via a puncture of the caudal vein. Immediately, samples were processed for the MN test as blood smears (5 µL) on glass slides and left for air dryness. Samples of gill, liver and muscle tissues were quickly and carefully taken, washed in normal saline (0.7% NaCl) for histopathological analysis.

Fish erythrocytes were used to monitor the genotoxic potency by investigating micronuclei (MN) and nuclear abnormalities (NA) appearances following the treatments. In brief, fixation of dry smeared samples was done in absolute methanol for 10 min then staining with Hematoxylin (15 min) and Eosin (15 min) was performed. Counting about 2000 cells per fish was used to analyze the mean percent of MN and NA. Kidney-shaped, budding and binucleated cells were counted as NA.

The histopathological alterations were evaluated in gill, liver and muscle tissues. Samples were fixed in 10% neutral formalin for 24 h, washed overnight in running tap water, and then rinsed in distilled water. The fixed organs were dehydrated in a gradual series of ethanol (70%–100%), placed in 2 changes of xylene, and then embedded in paraplast paraffin (56–58°C). The paraffin sections were cut (about 5 μm) using a rotary microtome and then were put on clean slides on a digital hot plate at 40°C for section spreading and water evaporation. A routine histology study was done on paraffin sections stained by Haematoxylene and Eosin (H&E) staining method (Suvarna et al. 2018).

The method of Finney (1952) for evaluating the dose-mortality response was followed using Probit and the fit goodness evaluation was performed by applying the *Chi*-square (χ^2^) test using the IBM-SPSS software version 21.1 (USA.( The normality test, Shapiro–Wilk’s, and Levene’s tests for variance homogeneity were used. One-way analysis of variance (ANOVA) was used to assess the normal data distribution and variance homogeneity. The significance was considered at *p* < 0.05.

## Results

**Fig. 1:**
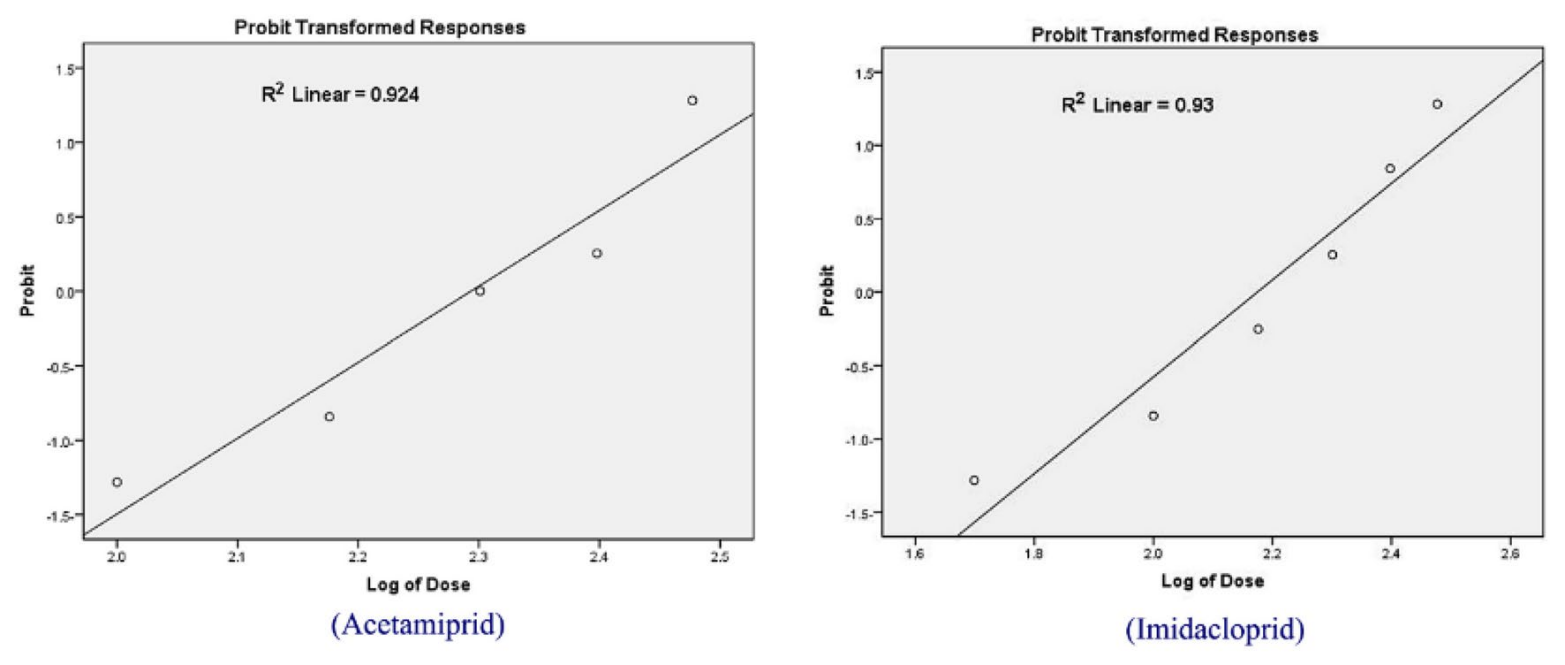
Logarithmic concentration–Probit line for determination of 96 and 72 h-LC_50_ of acetamiprid and imidacloprid respectively on *O. niloticus*

The LC_50_ was assessed in this study by the Probit method using the serial concentration responses (Fig. 1). The LC_50_ concentrations of Aceta and Imid were calculated from Probit regression equations. Probit model of acetamiprid (Probit (*p*) = − 13.404 + 5.849X, χ^2^ = 2.678, df = 5, Confident interval (CI), 95% = 163.262–228.954, *p* = 0.749) and imidacloprid (Probit (*p*) = − 8.327 + 3.823X, χ^2^ = 3.110, df = 5, CI (95%) = 115.043–186.751, *p* = 0.683) showed a good regression fit (R^2^: 0.924 for Aceta-96 h and R^2^: 0.93 for mid-72 h). Based on the LC_50_ of Aceta and Imid, which were 195.813 and 150.763 ppm respectively, the acute toxicity towards the *O. niloticus* of both insecticides did not show any significant differences (*p* < 0.05).

**Fig. 2:**
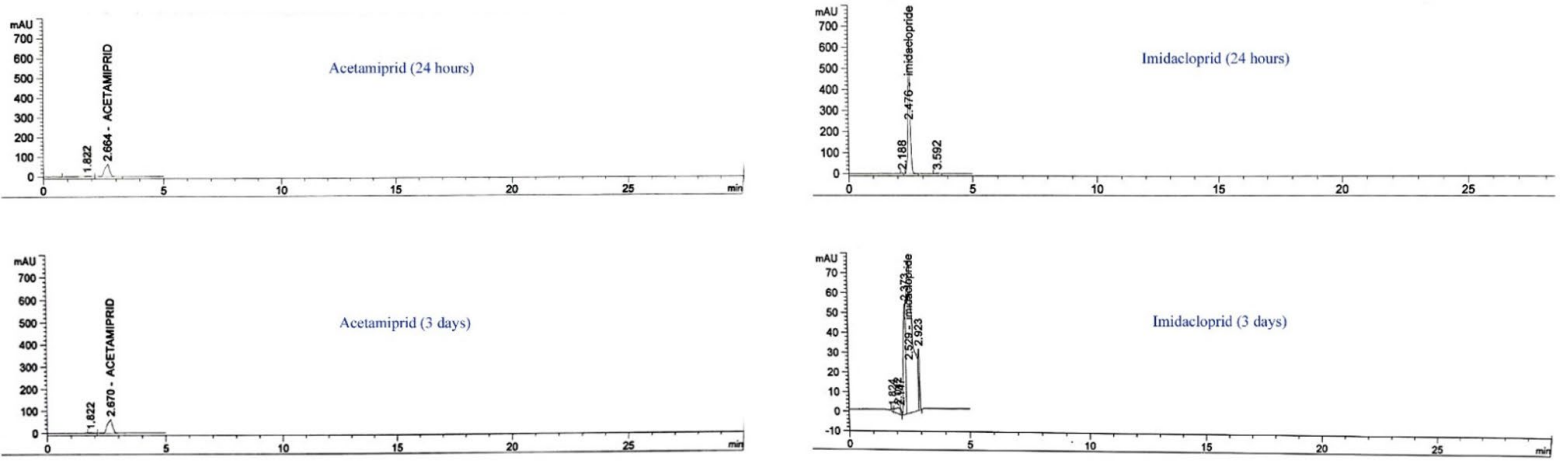
HPLC chromatogram shows the concentration of acetamiprid and imidacloprid after one and 3 days in aquaria water. The unchanged concentration of Aceta was observed, however, Imid showed high degradation only after 3 days

For the assessment of the stability of treatments in water, High Performance Liquid Chromatography (HPLC) analysis was performed. By detecting the measured concentration of tested compounds, results of the HPLC analysis, as shown in Fig. 2, revealed that there was no change in the nominal concentrations of Aceta or Imid in water after 24 h. However, after 3 days, Aceta showed a measured concentration equal to the nominal one (100% stability) and Imid had a measured value of ~ 35%. Based on these results, a 24 h static renewal protocol was utilized to ensure consistent exposure.

**Fig. 3:**
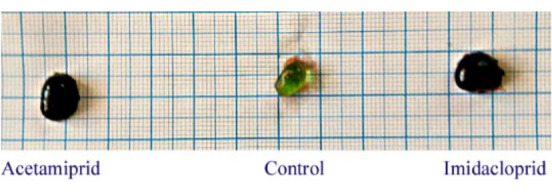
Representative photograph of *O. niloticus* gall bladders showing the effect induced by acetamiprid (19.5 ppm) and imidacloprid (15 ppm) exposure for 21 days

There was no mortality throughout the experiment (Aceta, 19.5 ppm and Imid, 15 ppm). However, in neonicotinoid-treated groups, general color darkening, sluggish swimming, raised fins, and lethargy were noted. Enlarged dark gall bladders were also recorded compared with controls among these groups (Fig. 3).

**Fig. 4:**
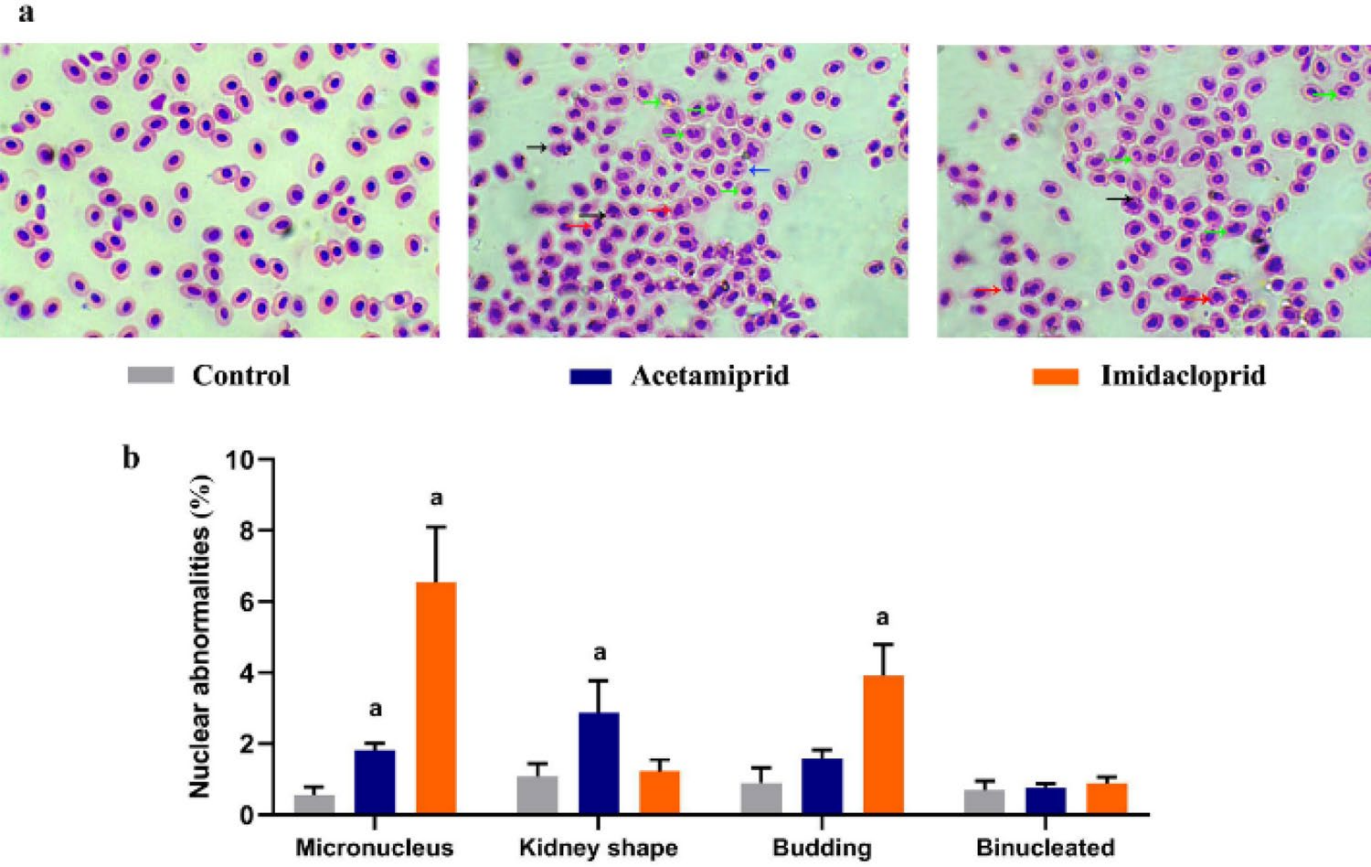
Erythrocytic nuclear abnormalities and micronuclei induced by acetamiprid (19.5 ppm) and imidacloprid (15 ppm) exposure on *O. niloticus* for 21 days. **A** H&E-stained representative field of erythrocytes shows nuclear abnormalities where, micronucleus (black arrow), kidney shape (green arrow), budding (blue arrow) and binucleated (red arrow). All data were shown as mean ± standard deviation (**B**), (n = 5). a reveals significance (*p* < 0.05) relative to control

Nuclear erythrocytic alterations and micronucleus (MN) appearance were assessed in this study to monitor the genotoxic effect of Aceta and Imid exposure (Fig. 4a). Fish exposed to Aceta and Imid revealed a significant (*p* < 0.05) elevated count of MN by ~ 2.2 and 10 folds respectively, when compared to control. However, relative to untreated fish, the kidney shape, budding and binucleated forms showed an elevation (*p* < 0.05) by 165.74%, 76.67% and 10.32% respectively in fish exposed to Aceta and by 13.89%, 334.44% and 26.07% respectively in Imid-exposed fish (Fig. 4b).

**Fig. 5:**
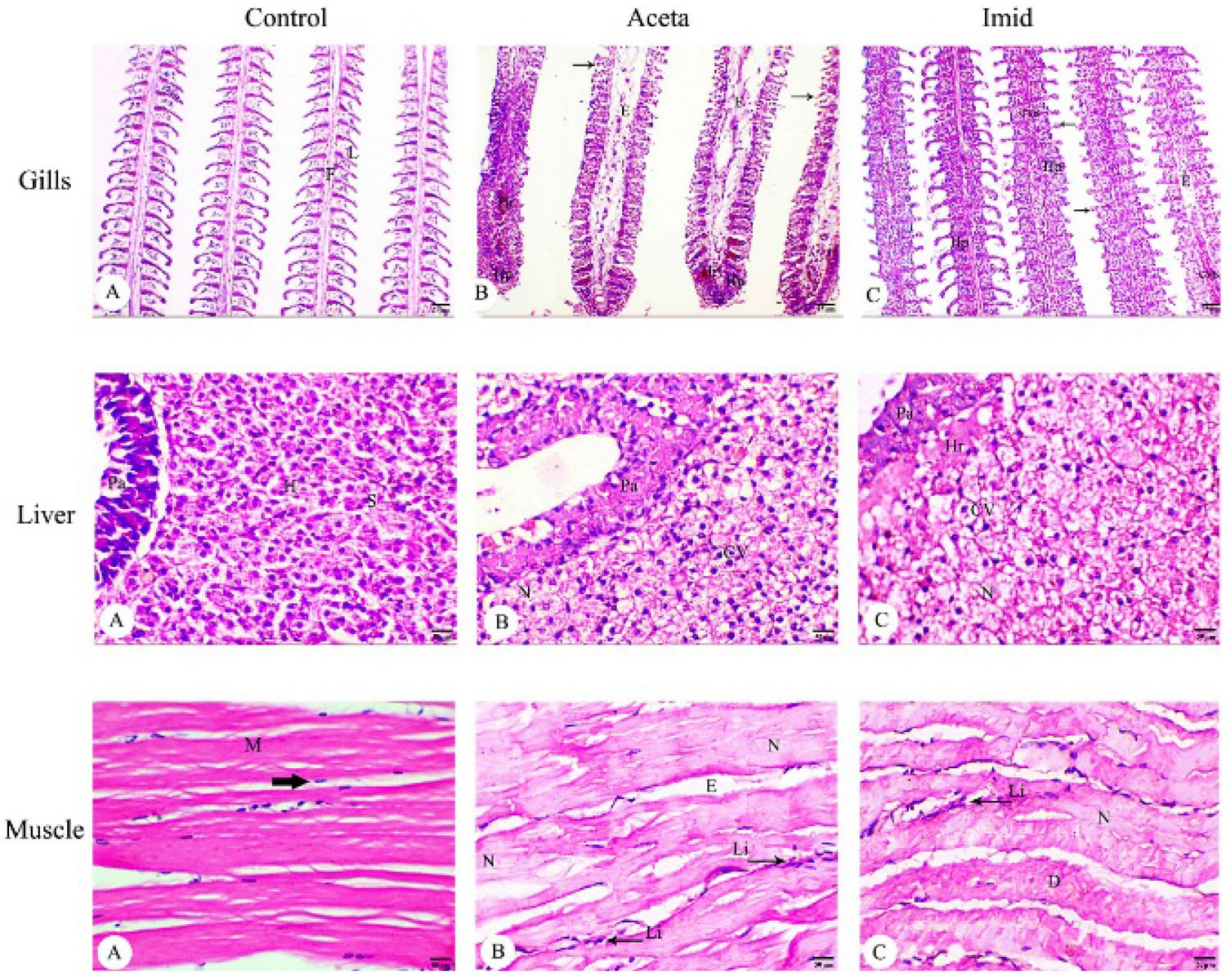
Light micrographs of the gill, liver and muscle of *Oreochromis niloticus* showing the effect of acetamiprid (Aceta, 19.5 ppm) and imidacloprid (Imid, 15 ppm) exposure. Where, **A** Control group, **B** Aceta group, and **C** Imid group. *F* filaments, *L* lamellae, Fusion of the secondary lamellae (Thin arrows), *Hp* hyperplasia, *Hr* hemorrhage, *E* edema, *H* hepatocytes, *Pa* pancreatic area, *S* sinusoid, *CV* cytoplasmic vacoulation, *cvs* central venous sinus, *M* Multinucleated fibers with peripheral nuclei (Thick arrow), *N* necrosis area, *D* degeneration, leucocytic infiltration (Li) and edema (E) between myomeres (H&E, Scale Bar: 25 µm (gill), 50 µm (liver & muscle)

The H&E-stained sections of gills, liver and muscles of exposed and control fish were evaluated to assess the toxicity of Aceta and Imid exposures (Fig. 5). The Nile tilapia gills from the untreated fish consist of four cartilaginous arches on each side of the buccal cavity. Each one is comprised of primary filaments with a double row of secondary lamellae which consists of a thin epithelium layer. Fish from the Aceta group (19.5 ppm) showed severe histopathological changes in their gills, which were edematous and suffered from hyperplasia, hemorrhage, and fusion of the secondary lamellae. Nile tilapia from the Imid-exposed group (15 ppm) exhibited a series of severe histological changes which were noticed in gill sections, such as the dilatation of congested capillaries, central venous sinus in filaments, a high thickening of the epithelium of gill lamellae and hyperplasia (Fig. 5). Moreover, in the case of control liver sections, they consist of hepatocytes arranged in branched laminae, and separated by blood sinusoids. Hepatocytes are polygonal liver cells with a homogenous cytoplasm and a spherical nucleus. The pancreatic structure which is associated with venous vessels was also observed. Nile tilapia fish from the Aceta-exposed group (19.5 ppm) exhibited a degeneration in hepatic and pancreatic structures, necrosis, and cytoplasmic vacuolation in hepatocytes. Similarly, more severe histopathological changes and haemorrhage were observed in the hepatic parenchyma of Imid-exposed group (15 ppm) (Fig. 5). Muscles of Nile tilapia from untreated fish showed the muscle layers formed from muscle fibers (myomeres) with a typical morphologic pattern of multinucleated fibers and peripheral nuclei. However, in the Aceta and Imid groups, suffering from the degeneration and necrosis in myomeres, associated with leucocytic infiltration and edema between myomeres was observed with more severity in the Imid-exposed group (Fig. 5).

## Discussion

Chemicals that contaminate any system have the probability of altering the structure and function of the community (Saeed et al. 2014; El-Garawani et al. 2021a). Neonicotinoid insecticides have a potential for leaching, drainage, run-off, or snowmelt (Berheim et al. 2019). A variety of these insecticides have been found in water bodies such as tanks, lakes, groundwater, and streams (Ansoar-Rodríguez et al. 2016; Hladik et al. 2018). Diverse effects on feeding, movement, immunity, growth, and development were noticed in aquatic organisms after neonicotinoids' exposure (Hayasaka et al. 2012; Nyman et al. 2013).

Neonicotinoids have a wide range of half-lives in the environment, ranging from a few minutes to many weeks in water (Anderson et al. 2015; Juan García 2021). Previous studies showed that the half-life time of acetamiprid in water was recorded as 4.7 days (Sánchez-Bayo and Hyne 2014; Zoumenou et al. 2019). However, for imidacloprid, the half-life time in water was estimated at 3 days (Redlich et al. 2007). This is concurrent with our results of HPLC as acetamiprid showed stability without degradation up to 3 days and imidacloprid degraded after 3 days by about 65%.

In this study, a significant increase in nuclear abnormalities in Aceta and Imid-exposed groups was observed. The loss of a chromosomal part or the whole during cell division usually leads to micronucleus (MN) formation which is considered as a genotoxicity indicator (Schmid 1975; Omar et al. 2021). Clastogenic agents may cause these abnormalities by altering the integrity of DNA leading to chromosomal fragmentation or modifying mitosis and causing cell spindle breakdown as aneugenic agents (Abdel-Khalek et al. 2020). Herein, the high MN and NA formation suggested that the affected hematopoietic tissues which may be caused either by breaks in DNA strands or dysfunction of the mitotic spindle (Alimba et al., 2017; Hathout et al., 2021). In parallel to our results, there was an increase in the MN and NAs frequencies in the Nile tilapia with various neonicotinoids' exposure (Vieira et al. 2018; Abdel-Khalek et al. 2020). Oxidative stress and free radical generation could affect macromolecules such as nucleic acids, proteins and lipids leading to cell damage and even death (Murakami et al. 2020).

Fish gills are frequently used as bioindicators reflecting water quality and the effects of xenobiotic exposure due to their susceptible structure and direct contact with water (Mallatt 1985; Paulino et al. 2012). In agreement with the previous studies, the Aceta-exposed fish, herein, showed hyperplasia, fusion of secondary lamellae and edematous forms in gills (Figueiredo-Fernandes et al. 2007; Yoon et al. 2015; Nofal et al. 2019; Ghayyur et al. 2020, 2021). Furthermore, imid-exposed fish showed the epithelial thickening of gill lamellae and dilatation of congested capillaries (Visoottiviseth et al. 1999). Gill hyperplasia was observed too (Javed et al. 2016; Shan et al. 2020; Günal et al. 2020). It can be argued that gill epithelium was the principal entry point of contamination which would multiply, thereby causing hyperplasia (Javed et al. 2016). The hyperplasic changes such as epithelial hyperplasia and lamellar fusion are nonspecific defence responses that protect the organism from the increased up-take of various xenobiotics by maximizing the distance between blood vessels and toxicants (Xu et al. 2014; Günal et al. 2020).

Similar to our findings, the liver of *C. auratus* exposed to Chromium showed hemorrhage which may have resulted due to the pressure of blood and congested blood vessels caused by toxicant exposure (Velma and Tchounwou 2010). Further, cytoplasmic vacuolation and necrosis were noticed among Imid and Aceta-exposed liver sections. These findings were in agreement with Visoottiviseth et al. (1999); Figueiredo-Fernandes et al. (2007) who studied the effect of water borne copper and Triphenytin Hydroxide exposure on the liver of Nile tilapia. In parallel to our findings, concentrations of Imid exerted hydropic degeneration and vacuolization of hepatocytes (Günal et al. 2020) and hydropic degeneration and pyknotic nuclei (Ansoar-Rodríguez et al. 2016) in the liver of Nile tilapia. Similarly, concentrations of Aceta showed necrotic areas and vacuolation in the *O. niloticus* liver. Hepatic and pancreatic degeneration may occur due to the accumulation of leucocytes (Javed et al. 2016). However, the hydropic degeneration is due to the accumulation of water and electrolytes inside the cell (Ansoar-Rodríguez et al. 2016). Liver cell injury has been identified by many mechanisms (Grattagliano et al. 2002; Lee 2003) such as mitochondrial dysfunction, inhibition of fatty acid beta-oxidation and/or inhibition of respiratory enzymes or by a direct effect on mitochondrial DNA. The accumulation of lactate and reactive oxygen species may occur because free fatty acids are not metabolized. These radicals cause mitochondrial DNA damage leading to the breakdown of sinusoidal structures and pooling of blood in the liver through these mechanisms and eventually severe histopathological changes.

Neonicotinoids have a diverse effects on liver which affect the detoxification, enzyme production and metabolism (Sharma et al. 2019). Further, they caused alterations in gills that are considered a chief organ of pollutants' targeting (Macirella et al. 2019). These effects could eventually harm the physiological status of the body including growth and muscles' components which is almost at risk of being harmed by various types of pollutants (Haredi et al. 2020). In this study, Aceta and Imid-exposed fish showed histopathological alterations in muscles such as degeneration, focal areas of necrosis and edema (Kaoud and El-Dahshan 2010), and leucocytic infiltration (Haredi et al. 2020). These histopathological alterations could be due to the oxidative stress and free radical generation induced by acetamiprid and imidacloprid exposure (Monteiro et al. 2006) leading to damage in the structure of muscle fibers (Long et al. 2022). Reactive oxygen species (ROS) may interact with susceptible biological macromolecules, causing lipid peroxidation (LPO), DNA damage, and protein oxidation which eventually lead to oxidative stress (Livingstone 2001). In the future, in-depth assessment of the neonicotinoids' potential hazard to aquatic organisms should be conducted over various concentrations and other neonicotinoids to generalize the findings and to investigate the underlying mechanism responsible for these adverse effects.

The current study indicates that acetamiprid and imidacloprid were genotoxic as assessed by erythrocytic nuclear abnormalities and histological adverse effects on gills, liver, and muscles. Imid showed more severity among the investigated issues than Aceta. Therefore, this study is important for *O. niloticus* due to its economic value and the effect on the quality and safety of human consumption.
